# Two Missense Variants Detected in Breast Cancer Probands Preventing BRCA2-PALB2 Protein Interaction

**DOI:** 10.3389/fonc.2018.00480

**Published:** 2018-10-25

**Authors:** Laura Caleca, Irene Catucci, Gisella Figlioli, Loris De Cecco, Tina Pesaran, Maggie Ward, Sara Volorio, Anna Falanga, Marina Marchetti, Maria Iascone, Carlo Tondini, Alberto Zambelli, Jacopo Azzollini, Siranoush Manoukian, Paolo Radice, Paolo Peterlongo

**Affiliations:** ^1^Unit of Molecular Bases of Genetic Risk and Genetic Testing, Department of Research, Fondazione IRCCS Istituto Nazionale dei Tumori, Milan, Italy; ^2^Genome Diagnostics Program, IFOM the FIRC Institute of Molecular Oncology, Milan, Italy; ^3^Platform of Integrated Biology, Department of Applied Research and Technology Development, Fondazione IRCCS Istituto Nazionale dei Tumori, Milan, Italy; ^4^Ambry Genetics, Department of Clinical Diagnostics, Aliso Viejo, CA, United States; ^5^Cancer Outreach and Risk Assessment, Via Christi Hospitals, Wichita, KS, United States; ^6^IFOM, Fondazione Istituto FIRC di Oncologia Molecolare, Milan, Italy; ^7^Cogentech Cancer Genetics Test Laboratory, Milan, Italy; ^8^Department of Immunohematology and Transfusion Medicine, Azienda Ospedaliera Papa Giovanni XXIII, Bergamo, Italy; ^9^USSD Laboratorio Genetica Medica, Azienda Ospedaliera Papa Giovanni XXIII, Bergamo, Italy; ^10^Unit of Medical Oncology, Azienda Ospedaliera Papa Giovanni XXIII, Bergamo, Italy; ^11^Unit of Medical Genetics, Department of Medical Oncology and Hematology, Fondazione IRCCS Istituto Nazionale dei Tumori, Milan, Italy

**Keywords:** breast cancer, breast cancer predisposition genes, PALB2, BRCA2, VUS, functional analyses, PALB2-BRCA2 interacting domain

## Abstract

PALB2 (partner and localizer of BRCA2) was initially identified as a binding partner of BRCA2. It interacts also with BRCA1 forming a complex promoting DNA repair by homologous recombination. Germline pathogenic variants in *BRCA1, BRCA2* and *PALB2* DNA repair genes are associated with high risk of developing breast cancer. Mutation screening in these breast cancer predisposition genes is routinely performed and allows the identification of individuals who carry pathogenic variants and are at risk of developing the disease. However, variants of uncertain significance (VUSs) are often detected and establishing their pathogenicity and clinical relevance remains a central challenge for the risk assessment of the carriers and the clinical decision-making process. Many of these VUSs are missense variants leading to single amino acid substitutions, whose impact on protein function is uncertain. Typically, VUSs are rare and due to the limited genetic, clinical, and pathological data the multifactorial approaches used for classification cannot be applied. Thus, these variants can only be characterized through functional analyses comparing their effect with that of normal and mutant gene products used as positive and negative controls. The two missense variants *BRCA2*:c.91T >G (p.Trp31Gly) and *PALB2*:c.3262C >T (p.Pro1088Ser) were detected in two breast cancer probands originally ascertained at Breast Cancer Units of Institutes located in Milan and Bergamo (Northern Italy), respectively. These variants were located in the BRCA2-PALB2 interacting domains, were predicted to be deleterious by *in silico* analyses, and were very rare and clinically not classified. Therefore, we initiate to study their functional effect by exploiting a green fluorescent protein (GFP)-reassembly *in vitro* assay specifically designed to test the BRCA2-PALB2 interaction. This functional assay proved to be easy to develop, robust and reliable. It also allows testing variants located in different genes. Results from these functional analyses showed that the *BRCA2*:p.Trp31Gly and the *PALB2*:p.Pro1088Ser prevented the BRCA2-PALB2 binding. While caution is warranted when the interpretation of the clinical significance of rare VUSs is based on functional studies only, our data provide initial evidences in favor of the possibility that these variants are pathogenic.

## Introduction

Approximately 20% of the familial aggregation of breast cancer is related to the presence of germline pathogenic variants in the tumor suppressor high-risk genes *BRCA1* (MIM#113705) and *BRCA2* (MIM#600185) [reviewed in (1)]. Additional germline variants in several other genes, including *PALB2* (partner and localizer of BRCA2) (MIM#610355) have also been implicated in increased predisposition to breast cancer (2, 3). Estimated cumulative breast cancer risk by age of 70 conferred by pathogenic variants in *BRCA1* and *BRCA2* is approximately 60 and 50%, respectively (4, 5). Loss of function *PALB2* pathogenic variants confer a breast cancer risk of 35% by age of 70, that is comparable to that conferred by *BRCA2* pathogenic variants (6). Sequencing of these genes has become a key step of the clinical management of breast cancer families as the carriers of a pathogenic variants may be offered appropriate surveillance programs or risk reducing options, whereas the non-carriers may be advised to follow the same recommendations offered to the general population (7).

The clinical utility and efficacy of genetic testing rely on the possibility to establish a correlation between the detected genetic variant and its protein functional effect. As an example, pathogenicity is generally inferred for variants introducing premature termination codons (PTCs), or affecting mRNA integrity and/or stability that give rise to functionally compromised proteins. However, the assessment of the clinical relevance of other variants, especially those that are rare, may not be equally straightforward. These are referred to as variants of uncertain significance (VUSs) and typically include missense variants, small in-frame deletions or insertions, exonic and intronic alterations potentially affecting the mRNA splicing, and variants in regulatory sequences (4, 8). Many of such variants located in the *BRCA1, BRCA2*, and *PALB2* genes have been deposited as “unclassified” in publicly available databases. The current approach to clinically classify a VUS is the multifactorial likelihood prediction model in which, data from epidemiological, genetic, pathological and clinical analyses are combined in order to derive a posterior likelihood of pathogenicity. However, reaching odds ratios in favor of or against causality requires such analyses to be based on several independent observations or to be carried out in large sample series which are usually difficult to obtain if a variant is rare (9, 10). This provides a compelling rationale to the inclusion in the multifactorial model of additional experimental evidences. As a possibility, VUSs —especially those located in the coding regions—can be studied using *in vitro* and functional assays that compare the effect of normal and mutant gene products.

At the molecular level, PALB2 was identified as a binding partner of BRCA2 and was subsequently shown to bridge, via direct protein-protein interaction, BRCA1 and BRCA2 at sites of DNA damage (11–13). Here, this complex promotes the repair by homologous recombination (HR) of the highly genotoxic DNA lesions, such as double-strand breaks (DSBs) or inter-strand crosslinks (ICLs) (14, 15). These BRCA1-PALB2-BRCA2 interactions are mediated via the coiled-coil domains located at the N-terminus of PALB2 (amino acids 9-44) and at the C-terminus of BRCA1 (amino acids 1,393–1,424), and by the seven-bladed β-propeller WD40 (tryptophan-aspartic acid rich) domain of the C-terminal end of PALB2 (amino acids 836–1,186) binding a domain in the N-terminal end of the BRCA2 (amino acids 21–39) (16, 17). Functional assays based on these domain bindings were used to study patient-derived missense variants in *BRCA1* and *BRCA2* to provide evidence in favor of or against pathogenicity. Three *BRCA2* missense variants, the c.73G>A (p.Gly25Arg), c.91T>C (p.Trp31Arg), and c.93G>T (p.Trp31Cys) were found to disrupt the BRCA2-PALB2 interaction, causing deficiencies in BRCA2 localization to the nucleus and in HR mediated DSB repair (16). Similarly, three *BRCA1* missense variants, the c.4198A>G (p.Met1400Val), c.4220T>C (p.Leu1407Pro), and c.4232T>C (p.Met1411Thr) abrogated or moderately impaired the BRCA1-PALB2 binding, causing reduced HR activity (17, 18). To date, only few patient-derived missense variants in the *PALB2* gene have been investigated for pathogenicity. Among these, the *PALB2*:c.104T>C (p.Leu35Pro), located in the coiled-coil domain, was found to co-segregate with two breast cancer cases in a family with a strong history for the disease, and was shown to abrogate the BRCA1-PALB2 binding and to completely prevent HR and resistance to DNA damaging agents. As a result, the p.Leu35Pro was suggested to be a pathogenic variant (19) and is to our knowledge the sole variant in *PALB2* to date suggested to be pathogenic. All these findings emphasize that functional assays on VUS located in the BRCA1-PALB2-BRCA2 interaction domains may provide clues on their pathogenicity and that other variants affecting such interactions may be associated with breast cancer susceptibility.

In the current study, we aimed to characterize functionally the two rare missense variants, *PALB2*:c.3262C>T (p.Pro1088Ser) and *BRCA2*:c.91T>G (p.Trp31Gly), that were initially identified in breast cancer families and that are located in the protein interaction domains. These two variants were tested for pathogenicity using the green fluorescent protein (GFP)-reassembly *in vitro* assay that was recently developed for the study of protein-protein interactions (20, 21).

## Patients and methods

### Breast cancer probands and genotyping analysis

The two female Italian breast cancer probands included in this study were originally considered eligible for clinical genetic testing in breast cancer genes, based on criteria including age of onset for breast cancer and family history for the disease. One proband, recruited at the Genetics Unit of Fondazione IRCCS Istituto Nazionale dei Tumori in Milan (INT), was tested for mutations in the coding regions of *BRCA1* and *BRCA2* by massively parallel sequencing, using TruSeq Custom Amplicon v.1.2 (Illumina), and multiplex ligation-dependent probe amplification (MLPA) resulting carrier of the *BRCA2*:p.Trp31Gly. These tests were performed at Cogentech Cancer Genetic Test Laboratory (CGT Lab). The other proband, recruited at the Unit of Medical Oncology of the Ospedale Papa Giovanni XXIII in Bergamo (HPG23), was tested for mutations in the coding regions of *BRCA1* and *BRCA2* by Sanger sequencing and MLPA at Cogentech CGT Lab. No *BRCA1* or *BRCA2* mutations were detected, and so this probands was tested at Laboratorio Genetica Medica, HPG23 by massively parallel sequencing using TruSight Cancer assay (Illumina). No pathogenic or likely pathogenic variants were found and the only deleterious variant detected was the missense *PALB2*:p.Pro1088Ser variant.

Genotyping of the *PALB2*:p.Pro1088Ser was performed using a custom TaqMan assay (probes and experimental conditions are available upon request). This variant was tested in familial and consecutive breast cancer cases ascertained at HPG23, and in female blood donors used as controls recruited at the AVIS Bergamo.

All individuals included in this study and herein described signed an informed consent to the use of their biological samples and clinical data for research project. This study was approved by Ethical Committee of Fondazione IRCCS Istituto Nazionale dei Tumori, Milan, and Ethical Committee of the Province of Bergamo.

### Positive/negative controls for the GFP-reassembly *in vitro* assay

Six *BRCA2* or *PALB2* variants located in the protein interaction domain were included as positive and negative controls. The *PALB2*:c.2816T>G (p.Leu939Trp) variant was reported to be not associated with breast cancer risk and to not alter the protein DNA repair activity by HR (22, 23). The *BRCA2*:c.79A>G (p.Ile27Val) and *PALB2*:c.3064AT>GC (p.Met1022Ala) missense variants were functionally tested and resulted not disrupting the BRCA2-PALB2 interaction (16, 24). These three variants were used as positive controls. The three missense variants *BRCA2*:c.93G>T (p.Trp31Cys), *BRCA2*:c.91T>C (p.Trp31Arg) and *PALB2*:c. 3073G>A (p.Ala1025Arg) were reported to functionally prevent the BRCA2-PALB2 binding and were used as negative controls (16, 24). All of these variants were patient-derived with the exception of the *PALB2*:p.Met1022Ala and *PALB2*:p.Ala1025Arg that were synthetically designed based on crystallography analyses.

### Plasmid construction

The pET11a-NfrGFP-Z and pMRBAD-Z-CfrGFP expression vectors, encoding anti-parallel leucine zipper motifs (Z) fused to the N-terminal or C-terminal fragment of the GFP Protein (NfrGFP and CfrGFP, respectively) (20) were kindly donated by TJ Magliery from the Ohio State University in Columbus (OH, USA). The DNA fragments encoding the N-terminal end of BRCA2 (amino acids 10–40) and the WD40 domain of PALB2 (amino acids 836-1186) were amplified from the cDNA of the 293T cells by PCR. The purified PCR products were subcloned into pET11a-NfrGFP between *XhoI* and *BamHI* restriction sites and pMRBAD-Z-CfrGFP between *NcoI* and *AatII* restriction sites, replacing the fragments encoding Z motifs. The *BRCA2* c.79A>G (p.Ile27Val), c.91T>C (p.Trp31Arg), c.91T>G (p.Trp31Gly), c.93G>T (p.Trp31Cys) and the *PALB2* c.2816T>G (p.Leu939Trp), c.3073G>A (p.Ala1025Arg), c.3266C>T (p.Pro1088Ser) variants were obtained by direct mutagenesis of pET11a-NfrGFP-BRCA2 and of pMRBAD-PALB2-CfrGFP using the QuickChange XL Site-directed Mutagenesis Kit (Stratagene) according to the manufacturer's instruction. The *PALB2* c.3064AT>GC (p.Met1022Ala) was obtained by the overlap extension PCR mutagenesis method (25). The presence of variants in recombinant clones was verified by DNA sequencing (Eurofins Genomics).

### GFP-fragment reassembly screening

Compatible pairs of plasmids (pET11a-NfrGFP-BRCA2 and pMRBAD-PALB2-CfrGFP, both as wild-type and mutant forms) were co-transformed into BL21 (DE3) *E. coli* competent cells by electroporation. Single colonies were then picked and used to inoculate 2 ml of LB broth medium containing ampicillin (100 μg/ml) and kanamycin (35 μg/ml). Following overnight incubation at 37°C, the cultured cells were diluted 1:1,000 and 100 μl were plated on inducing LB agar (LBA) plates supplemented with 20 μM Isopropyl β-D-1-tiogalattopiranoside (IPTG) and 0.2% L-arabinose, to promote the expression of recombinant proteins. The plates were incubated at 30°C for 24 h and then 3 days at room temperature (RT). Fluorescence was observed after excitation with long-wave (365 nm) UV light in combination with the short pass (SP) emission filter using a Syngene image capture system (SYNGENE) as specified by the manufacturer.

### Purification of the reassembled GFP complexes

The pET11a-NfrGFP-BRCA2 (both wt and mutant forms) also encode a hexa histidine (H_6_)-tag at the N-terminus of the NfrGFP useful for rapid purification by Immobilized metal affinity chromatography (IMAC) method of the H_6_-tagged proteins. This method exploits the strong binding of H_6_-tagged protein to metal ions as nickel, allowing them to be separated from other proteins that have lower or no affinity.

Co-transformed bacterial cells were recovered from inducing LBA media using a plate spreader and resuspended in two 1 ml-aliquots of 1X phosphate buffered saline (PBS). After centrifugation, each pellet was resuspended in 50 μl of 1xSDS loading buffer (whole cell extracts), or in 1 ml of lysis buffer (50 mM Tris-HCl, 300 mM NaCl, 0.1% v/v Triton X-100, 100 μM EDTA pH8.0, 0.5 mg/ml lysozime, 20 mM Imidazole, protease inhibitors, 5 μg/ml DNase and RNase) for IMAC purification using the nickel nitrilotriacetic (Ni-NTA) agarose resin (QIAGEN), following the manufacturer's instructions. The purified protein complexes were subjected to 13% SDS-PAGE and visualized by Western blotting using a polyclonal anti-GFP antibody (#600-101-215; Rockland). Whole cell extracts were similarly resolved and visualized, to detect expression levels of the all NfrGFP-BRCA2 and CfrGFP-PALB2 fusion peptides.

## Results

### Identification of the *BRCA2*:c.91t>G (p.Trp31Gly) and the *PALB2*:c.3262C>T (p.Pro1088Ser) variants

**Figure 1:**
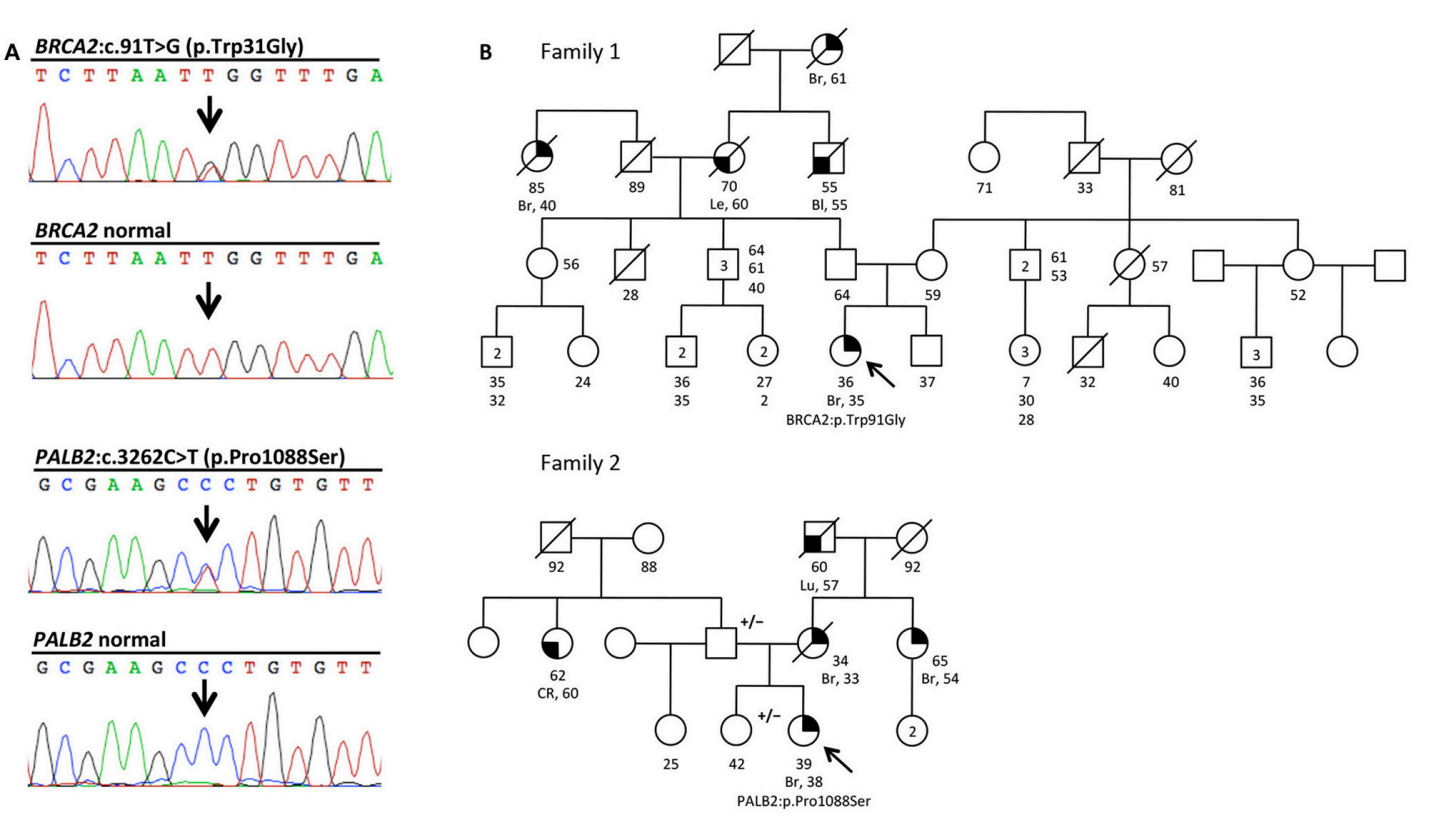
The *BRCA2*:c.91T>G (p.Trp31Gly) and the *PALB2*:c.3262C>T (p.Pro1088Ser) variants, and pedigrees of the two mutation carriers. **(A)** Electropherograms showing the *BRCA2* and *PALB2* sequence of the individuals carrying the two variants, and normal controls. **(B)** Family pedigrees 1 and 2 of the two Italians probands carrying the *BRCA2*:c.91T>G (p.Trp31Gly;) and the *PALB2*:c.3262C>T (p.Pro1088Ser) variants, respectively. Probands are indicated by arrow. Cancer type, age at diagnosis and age of death are reported when known. Age of healthy individuals, if known, was annotated at date of genetic counseling. Events occurred after genetic counseling, if known, are annotated. Additional relatives carrying the variants are indicated by +/–. Cancer type is reported as follows: Bl, bladder cancer; Br, breast cancer; CR, colorectal cancer; Le, leukemia; Lu, lung cancer.

**Figure 2:**
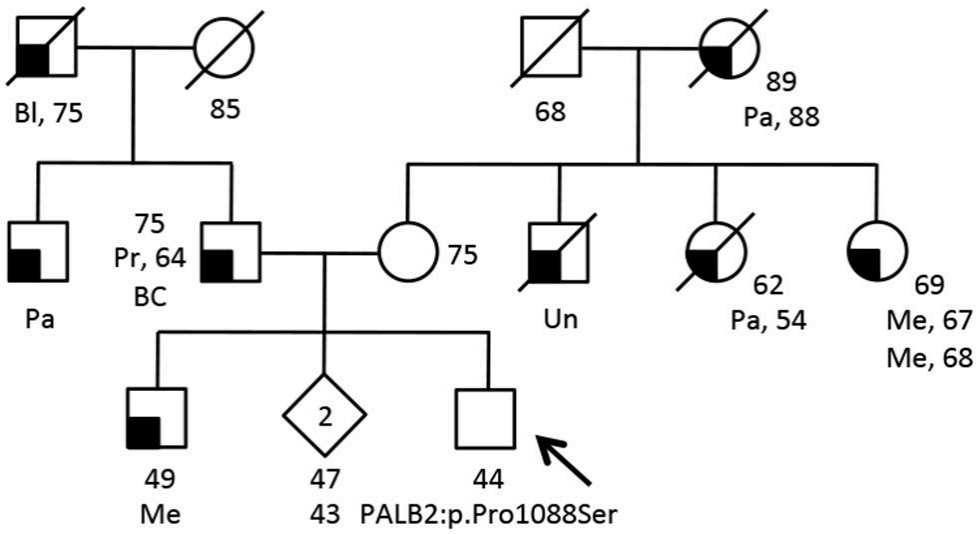
Family pedigree of the additional proband carrying the *PALB2*:c.3262C>T (p.Pro1088Ser) variant. The proband had large multi gene panel test (67 genes) due to a family history of cancer. The analysis was performed at the Ambry Genetics using Next Generation Sequencing and Array CGA (https://www.ambrygen.com/clinician/genetic-testing/28/oncology/cancernext-expanded). Proband is indicated by arrow. Cancer type, age at diagnosis and age of death are reported when known. Age of healthy individuals, if known, was annotated at date of genetic counseling. Events occurred after genetic counseling, if known, are annotated. Cancer type is reported as follows: BC, basal cells cancer; Bl, bladder cancer; Me, melanoma; Pa, pancreatic cancer; Pr, prostate cancer; Un, unknown.

Part of our research activity stems from the collaboration with several Breast Cancer Units in which clinical genetic testing is routinely performed. One of our major interest is to functionally study and characterize VUSs in breast cancer genes. In this study, we report the identification and describe the initial functional analyses of the *BRCA2*:p.Trp31Gly and the *PALB2*:p.Pro1088Ser variants. The *BRCA2*:p.Trp31Gly and the *PALB2*:p.Pro1088Ser variants were originally identified in two different Italian breast cancer probands born in Milano and Bergamo, respectively and are located in the BRCA2-PALB2 interacting domains. Both these probands developed breast cancer at a young age and reported a close relative affected with early onset breast cancer (≤ 40 years). Unfortunately, we were not able to ascertain other family members to be genotyped in order to attempt co-segregation analyses (Figure 1). None of these two variants were reported in public databases such as GnomAD and 1000 genomes. However, the *BRCA2*: < *underline* >p.Trp31Gly was annotated in ClinVar (https://www.ncbi.nlm.nih.gov/clinvar/) in a single individual submitted by Ambry Genetics and classified as a VUS. On the contrary, the *PALB2*:p.Pro1088Ser was not found in any of the clinical databases we searched. However, during the last annual meeting of the *PALB2* Interest Group (PIG; http://www.palb2.org/) colleagues from Ambry Genetics reported the finding of an additional carrier of the *PALB2*:p.Pro1088Ser variant. To our knowledge, to date, this is only the second proband found to carry this variant. For this reason, we report here his clinical phenotype and family cancer history. This proband was a male affected with colorectal polyps, type unknown, and from a family with cases of melanoma and pancreatic cancer but not breast cancers (Figure 2). Unfortunately, also in this case, no other samples were available for genotyping. We previously reported two different founder mutations, the *PALB2*:c.1027C>T (p.Gln343^*^) and the *BRCA2*:c.190T>C (p.Cys64Arg), originally identified in the Bergamo province where they have a carrier frequency approximately 10-fold higher than that of the Italian population (21, 26, 27). Hence, we genotyped the *PALB2*:p.Pro1088Ser in 126 familial and 477 consecutive breast cancer cases, and 1,074 controls all born in the province of Bergamo but no additional carriers were found.

### The *BRCA2*:c.91T>G (p.Trp31Gly) and the *PALB2*:c.3262C>T (p.Pro1088Ser) variants prevent the BRCA2-PALB2 binding

**Figure 3:**
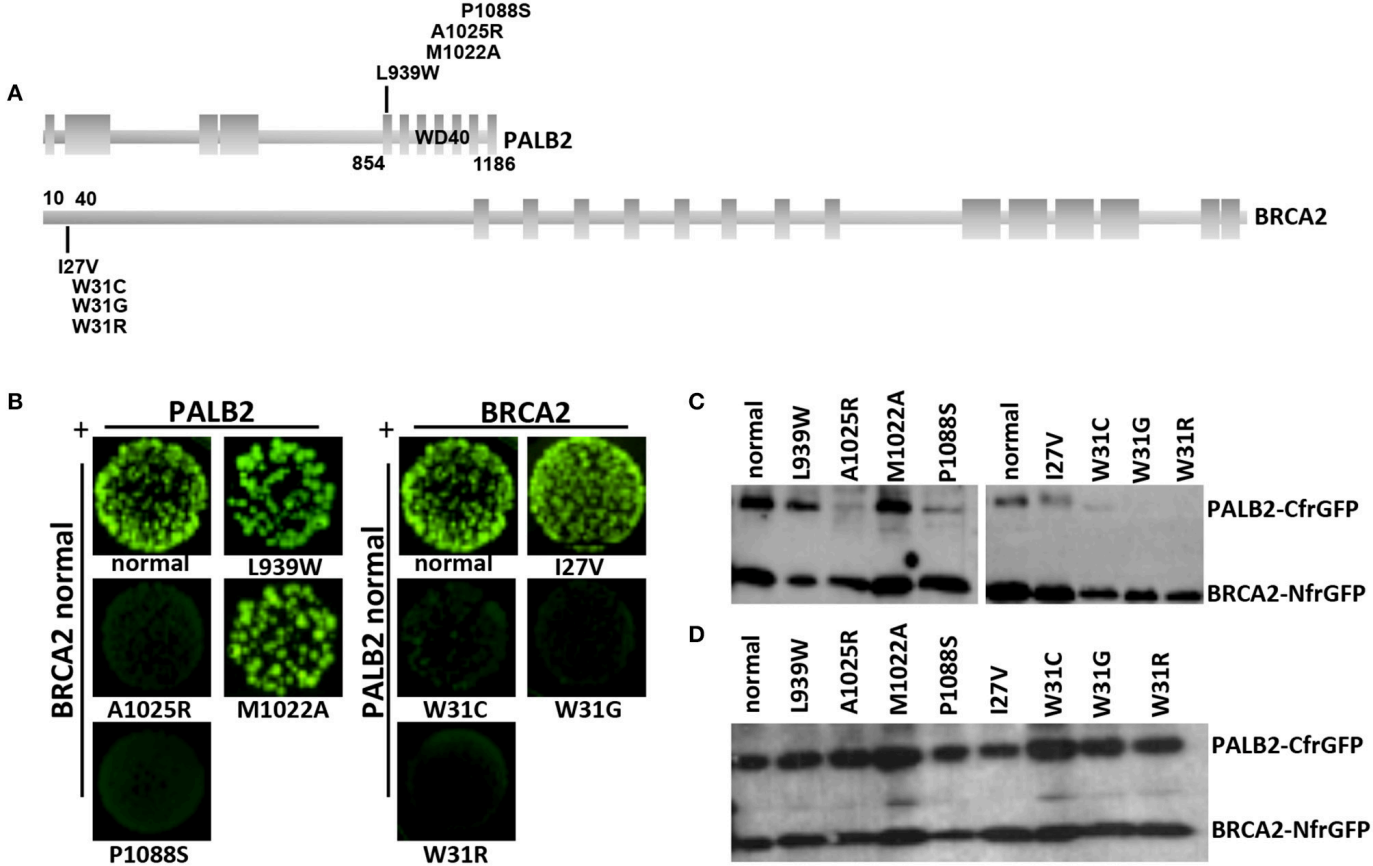
Detection of the BRCA2-PALB2 interaction. **(A)** Schematic representation of the specific domains mediating the BRCA2-PALB2 binding and of the location of variants analyzed in the present study. **(B)** GFP-reassembly *in vitro* assay. Fluorescence was recovered, under long-wave UV light (365 nm), after 24 h of growth at 30°C followed by 3 days of incubation at room temperature. The variants PALB2 L939W or M1022A and BRCA2 I27V were included as positive controls. The variants PALB2 A1025R and BRCA2 W31C or W31R were included as negative controls. **(C)** IMAC purification. The BRCA2-PALB2 reassembled complexes were purified from the co-transformed *E. coli* BL21 (DE3) cells, subjected to SDS-PAGE and visualized by Western blotting using a polyclonal anti-GFP antibody. **(D)** Analysis of expression of BRCA2-NfrGFP and PALB2-CfrGFP normal and mutant forms. Whole cell extracts from the co-transformed *E. coli* BL21 (DE3) cells were subjected to SDS-PAGE and visualized by Western blotting using a polyclonal anti-GFP antibody. Images of **(C, D)** were selected from original autoradiographic films shown in Supplementary Figure 1.

The *BRCA2*:c.91T>G and the *PALB2*:c.3262C>T variants were located within the protein domains mediating the BRCA2-PALB2 interaction (Figure 3A). To evaluate the effect of these variants on the BRCA2-PALB2 interaction, we exploited a bimolecular fluorescence complementation-based assay, the GFP-reassembly *in vitro* assay. In this assay, the GFP is dissected into two fragments, the N-terminal, NfrGFP and the C-terminal, CfrGFP which are fused to the N-terminal end of BRCA2 (amino acids 10-40) and the WD40 domain of PALB2 (amino acids 836-1186), respectively. These two plasmids are co-expressed in BL21 (DE3) *E. coli* cells and only if BRCA2-PALB2 interaction occurs, the GFP reassemble emitting cellular fluorescence after ultraviolet (UV) irradiation.

Bright GFP fluorescence was observed in bacterial cells co-expressing NfrGFP fused with normal BRCA2 and CfrGFP fused with either normal PALB2, the clinically neutral variant *PALB2*:p.Leu939Trp or *PALB2*:p.Met1022Ala (positive controls). Similar results were observed in bacterial cells co-expressing CfrGFP fused with normal PALB2 and NfrGFP fused with *BRCA2*:p.Ile27Val (positive control). On the contrary, no fluorescence was observed in bacterial cells co-expressing NfrGFP fused with normal BRCA2 and CfrGFP fused with either *PALB2*:p.Pro1088Ser, or *PALB2*:p.Ala1025Arg (negative control). Similar results were observed in bacterial cells co-expressing CfrGFP fused with normal PALB2 and NfrGFP fused with either *BRCA2*:p.Trp31Gly, or the negative controls *BRCA2*:p.Trp31Cys, or *BRCA2*:p.Trp31Arg (Figure 3B).

To confirm that GFP-reassembly was effectively due to the BRCA2-PALB2 interaction, the IMAC purified reassembled complexes were analyzed by Western blotting using a polyclonal anti-GFP antibody. Two bands corresponding to the components of the GFP reassembled complexes were detected in lysates of the bacterial cells that resulted fluorescent in the GFP-reassembly screening. On the contrary, no or low intensity bands corresponding to the PALB2-CfrGFP fused domains were observed in lysates from bacterial cells for whom no fluorescence was detected (Figure 3C). In general, any mutations can cause the decrease or the complete loss of expression of the encoded GFP fused peptides. Thus, we wanted to confirm that the lack or the low intensity of the PALB2-CfrGFP bands was not due to loss of expression. To this aim, the whole cell extracts were analyzed by Western blotting using a polyclonal anti-GFP antibody as previously described. In this experiment, we showed that normal and mutated fusion peptides were expressed to a similar extent indicating that the loss of fluorescence observed in the GFP-reassembly *in vitro* assay, was attributable to the lack of binding between the proteins and not to poor expression of the mutants (Figure 3D). All these results provided experimental evidence that both the *BRCA2*:p.Trp31Gly and the *PALB2*:p.Pro1088Ser variants abrogate the BRCA2-PALB2 binding.

## Discussion

In clinical settings, VUSs in breast cancer genes represent a serious issue in the process of disease risk assessment in carriers. Typically, results from different sources such as epidemiological, genetic, and clinical analyses are combined together in order to derive a posterior likelihood of pathogenicity used to classify a VUS. While this multifactorial approach is successful to classify common VUSs, variants that are rare or unique can only be studied through functional analyses.

In the present study, we investigated the pathogenicity of the two *BRCA2*:p.Trp31Gly and *PALB2*:p.Pro1088Ser variants performing functional analyses. The *BRCA2*:p.Trp31Gly was previously reported in a single proband and annotated as VUS. To our knowledge, the *PALB2*:p.Pro1088Ser was never detected before. Both variants are located in the interaction domains of BRCA2 and PALB2. Large part of the BRCA2 functions in the repair of the DNA double strand breaks and inter-strand crosslinks by HR depends from its interaction with PALB2. Thus, we developed a GFP-reassembly assay based on the testing of this interaction speculating that this binding assay would be a predictor of the effect of the variants on the BRCA2 integrity.

In the GFP-reassembly assay, we used six different *BRCA2* and *PALB2* missense variants as controls. Two patient-derived *BRCA2* variants, the p.Trp31Arg and p.Trp31Cys, and one synthetic *PALB2* variant, the p.Ala1025Arg, were known to prevent the BRCA2-PALB2 interaction. The patient derived *BRCA2*:p.Ile27Val and *PALB2*:Leu939Trp, and the synthetic *PALB2*:p.Met1022Ala were expected to not alter the binding of these proteins. For all of these variants, the results were concordant with their expected effect on the BRCA2-PALB2 binding.

The GFP-reassembly assay results indicated that both the *BRCA2*:p.Trp31Gly and *PALB2*:p.Pro1088Ser prevented the BRCA2-PALB2 interaction suggesting that in physiological conditions these alleles encode proteins that might be unable to interact with PALB2 and BRCA2, respectively. To our knowledge, the *PALB2*:p.Pro1088Ser is the first missense variant in the gene that was functionally shown to abrogate the binding with BRCA2. As the correct formation of the BRCA1-PALB2-BRCA2 complex is necessary for DNA repair by HR, our results provide evidences in favor of the hypothesis that the *BRCA2*:p.Trp31Gly and *PALB2*:p.Pro1088Ser are pathogenic variants. While this assumption is at present most likely—in example vs. the possibility that the variants are neutral—other aspects need to be considered for a clearer picture of the effect of these variants on breast cancer risk. Firstly, Foo and colleagues showed that both the *PALB2*:p.Leu35Pro and p.Tyr28Cys caused the loss of the interaction with BRCA1; however, only the p.Leu35Pro completely abrogated the HR activity and the p.Tyr28Cys caused a loss of approximately 65% of the HR activity (19). Hence, *PALB2* missense variants causing the loss of the binding with BRCA1 might confer different risk magnitude for breast cancer. To be conservative, we cannot exclude that this might be true as well for the PALB2 variants abrogating the binding with BRCA2. As a second point, caution should be taken when inferring on the nature of a missense variant on the bases of functional studies only. Park and colleagues reported that the *PALB2*:p.Leu939Trp variant might be pathogenic based on the fact that it resulted in altered BRCA2-PALB2 binding, decreased HR capacity, and increased sensitivity to ionizing radiation (28). However, we provided strong evidences deriving from additional functional studies and very large case-control studies that the *PALB2*:p.Leu939Trp is a neutral variant (23). As a final consideration, it should be noted that of the many missense variants that were functionally proved to prevent the BRCA1-PALB2-BRCA2 complex formation (16, 19, 24), all, with the only exception of the *PALB2*:p.Leu939Trp that is consider benign or likely benign, are annotated or should be treated clinically as VUS.

In conclusion, we report here results from functional studies indicating that the *BRCA2*:c.91T>G (p.Trp31Gly) and the *PALB2*:c.3262C>T (p.Pro1088Ser) missense variants abrogate the BRCA2-PALB2 protein binding. These data provide initial evidences corroborating the hypothesis that these variants are pathogenic. Importantly, novel data are warranted to progress in the clinical classification of these variants. The search for additional variant carriers and collection of their family members is crucial to provide genetic or pathological data; however, as the variants in study are very rare, we expect that not many variant carriers will be found in the near future. On the contrary, additional functional studies (i.e. testing specific protein functions in eukaryotic cells) could be immediately performed. While caution is warranted when clinical classification of a VUS is based on *in vitro* assays only, these results will provide additional evidences to better clarify the functional effect of the variants in study.

## Author contributions

PP and PR designed and supervised the study. TP, MW, SV, AF, MM, MI, CT, AZ, JA, and SM provided samples and data. LC, IC, and LD performed experiments. LC, IC, GF, PP, and PR analyzed data. LC, IC, PP, and PR wrote the manuscript. All authors contributing to, critically revised and approved the manuscript.

### Conflict of interest statement

TP is a full time paid employee of Ambry Genetics. The remaining authors declare that the research was conducted in the absence of any commercial or financial relationships that could be construed as a potential conflict of interest.
