# Supplementary material for: Two Missense Variants Detected in Breast Cancer Probands Preventing BRCA2-PALB2 Protein Interaction
Source: Front Oncol. 2018 Oct 25;8:480. doi: 10.3389/fonc.2018.00480 (PMC6210650; doi:10.3389/fonc.2018.00480)
Supplement: Supplementary file 1 [file Presentation_1.PPTX]

## Slide 1
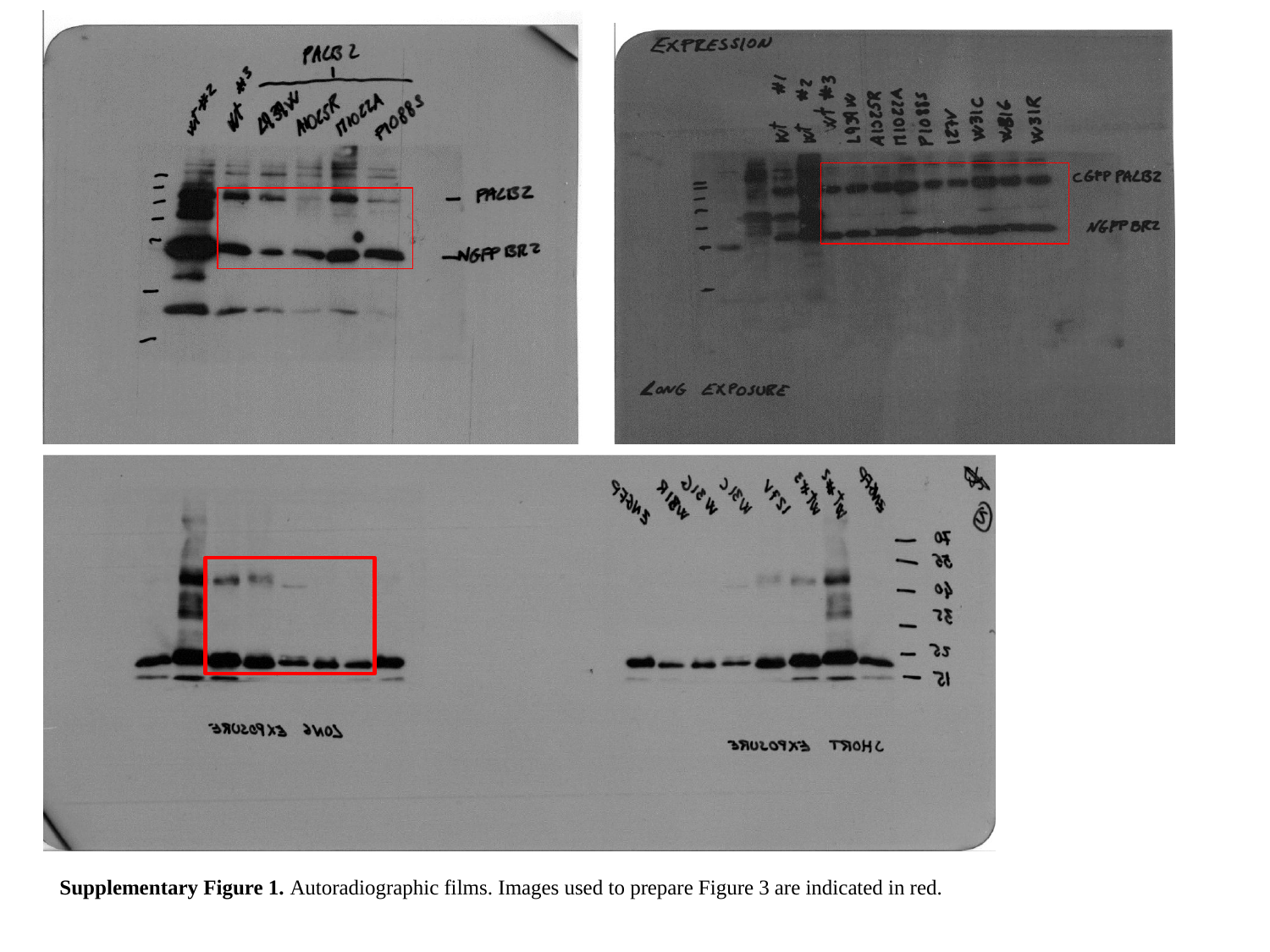

Supplementary Figure 1. Autoradiographic films. Images used to prepare Figure 3 are indicated in red.
